# Supplementary material for: The global geography of artificial intelligence in life science research
Source: Nat Commun. 2024 Sep 12;15:7527. doi: 10.1038/s41467-024-51714-x (PMC11392928; doi:10.1038/s41467-024-51714-x)
Supplement: Supplementary file 1 — Supplementary Information [file 41467_2024_51714_MOESM1_ESM.pdf]

The Global Geography of Artificial Intelligence in Life Science Research

Leo Schmallenbach[1], Till Bärnighausen[2,3,4], Marc J. Lerchenmueller[1,5]

[1] Department of Organization and Innovation, University of Mannheim, Mannheim, Germany
[2] Heidelberg Institute of Global Health (HIGH), Medical School, Heidelberg University, Heidelberg, Germany
[3] Harvard Center for Population and Development Studies, Harvard University, Cambridge, USA
[4] Africa Health Research Institute (AHRI), Somkhele and Durban, South Africa
[5] Leibniz Center for European Economic Research (ZEW), Mannheim, Germany

SUPPLEMENTARY MATERIAL

Table of Contents

S1 Review of Literature 2
S2 Number of AI-Related Life Science Articles by Country (2000-2022) 10
S3 Explanation of Concepts recorded in OpenAlex 14
S4 List of A* ranked Conference Proceedings 15
S5 List of AI-related Keywords used in Search Strategy 17
S6 Sample Creation and Accuracy 18
S7 AI life science research in journals versus conference proceedings 20
S8 References 32

S1 Review of Literature

Nr.	Reference	Main Findings
1	Abadi, H. H. N., He, Z., & Pecht, M. (2020). Artificial intelligence-related research funding by the US national science foundation and the national natural science foundation of China. *IEEE Access*, *8*, 183448-183459.	- The paper investigates funding by the U.S. National Science Foundation (NSF) and the National Natural Science Foundation of China (NSFC) from 2010 to 2019 in the field of artificial intelligence (AI).<br>- It compares the amount of funding and number of AI-related awards granted by the NSF and NSFC during the specified period.<br>- It identifies the key institutions and universities that received AI awards from the NSF and NSFC.<br>- The NSF granted a higher amount of funding and number of AI-related awards than the NSFC in the period of 2010-2019, the former granted approximately 2 billion in AI-related awards through 5,454 projects during this period.<br>- The study examines the key AI disciplines and applications of focus in the research funded by the NSF and NSFC.
2	AlShebli, B., Cheng, E., Waniek, M., Jagannathan, R., Hernández-Lagos, P., & Rahwan, T. (2022). Beijing's central role in global artificial intelligence research. *Scientific reports*, *12*(1), 21461.	- The paper analyses the inter-city collaborations and talent migrations in the field of AI using a dataset of 2.2 million AI papers.<br>- Identification of Beijing as an outlier, being the most productive since 2002, and housing the largest number of AI scientists since 1995.<br>- Beijing's role surpasses that of all other cities combined, making it the central gateway through which knowledge and talent flow from East to West in AI research.<br>- The top 20 cities, including Beijing, play a significant role in the rapid development of AI, attracting about 20% of global AI citations annually and producing 18 to 20% of AI papers worldwide.
3	Apell, P., & Eriksson, H. (2023). Artificial intelligence (AI) healthcare technology innovations: the current state and challenges from a life science industry perspective. *Technology Analysis & Strategic Management*, *35*(2), 179-193.	- The paper assesses the performance of the innovation system and identifies system-blocking mechanisms for AI healthcare technology innovations in the life science industry.<br>- It uses the Technological Innovation Systems (TIS) framework to analyze the structural and functional dynamics of AI healthcare technology innovations in West Sweden.<br>- It employs a mixed-method research approach, combining qualitative and quantitative data from secondary published sources and interviews with experts and life science business executives.<br>- The results highlight that limited resources and insufficient communication from healthcare professionals regarding their needs for improving healthcare using AI technology innovations are the main system weaknesses restricting innovation system performance.

		- The study suggests that policy interventions to increase available resources and formulate vision and mission statements to improve healthcare with AI technology innovations may enhance innovation system performance.
4	Bloom, N., Hassan, T. A., Kalyani, A., Lerner, J., & Tahoun, A. (2021). *The diffusion of disruptive technologies* (No. w28999). National Bureau of Economic Research.	- The paper utilizes the full text of millions of patents, job postings, and earnings conference calls to study the development and diffusion of disruptive technologies across various dimensions.<br>- The authors establish five stylized facts about the development and diffusion of disruptive technologies, including their spread across space, skill levels, and other dimensions.<br>- The analysis identifies 29 disruptive technologies - Artificial Intelligence is one of them - that had significant implications for businesses and jobs in the United States over the past two decades.<br>- The study shows that disruptive technologies typically emerge from a handful of urban areas, which house the majority of early patenting and employment in the technology before its commercial breakthrough.<br>- This pattern of "region broadening" is observed in virtually every technology examined, indicating the concentration of innovation in specific pioneer locations.
5	Bullock, J., Luccioni, A., Pham, K. H., Lam, C. S. N., & Luengo-Oroz, M. (2020). Mapping the landscape of artificial intelligence applications against COVID-19. *Journal of Artificial Intelligence Research*, *69*, 807-845.	- The paper identifies and categorizes various AI applications against COVID-19, including diagnosis and screening, drug discovery, epidemiology and public health, and social control and monitoring.<br>- AI has shown promise in accelerating the development of diagnostics, therapeutics, and vaccines for COVID-19.<br>- The paper discusses the challenges and limitations of AI applications in the context of COVID-19, such as data privacy concerns and the need for robust validation and regulation.<br>- It emphasizes the importance of interdisciplinary collaborations and the integration of AI with other technologies in the fight against COVID-19.<br>- The paper provides insights into the future directions and opportunities for AI in combating the pandemic, including the potential for AI to assist in early detection and prediction of outbreaks.
6	Cioffi, R., Travaglioni, M., Piscitelli, G., Petrillo, A., & De Felice, F. (2020). Artificial intelligence and machine learning applications in smart production: Progress, trends, and directions. *Sustainability*, *12*(2), 492.	- The paper aims to analyze the scientific literature on the application of artificial intelligence (AI) and machine learning (ML) in the manufacturing industry, specifically in the context of smart production.<br>- It conducted a literature review on ML and AI empirical studies published from 1999 to the present, highlighting the evolution of the topic before and after the introduction of Industry 4.0.<br>- The analysis included classification of the literature based on publication year, authors, scientific sector, country, institution, and keywords.<br>- The review identified 82 articles, with a greater number of works published by the USA and an increasing interest in AI and ML after the birth of Industry 4.0.

7	Claude, R., Charles-Daniel, A., Jean, A., & Jean-Francois, G. (2004). Bibliometric overview of the utilization of artificial neural networks in medicine and biology. *Scientometrics*, *59*, 117-130.	- The study provides an analysis of articles involving artificial neural networks (ANN) in medicine and biology, considering parameters such as the number of articles, impact factor, journal category, source country population, and gross domestic product.<br>- The top five countries with the most publications on artificial neural networks (ANN) in medicine and biology in 2004 were the USA, United Kingdom, Germany, Italy, and Canada. Other active countries in ANN research in these fields included Sweden, Netherlands, Spain, France, Japan, and China.<br>- The paper presents the distribution of ANN publications among subdisciplines of life sciences and clinical medicine.<br>- It highlights the impact of recent developments in biology, biotechnologies, and the growing research on ANN in biomedical sciences.
8	Davenport, T., & Kalakota, R. (2019). The potential for artificial intelligence in healthcare. *Future healthcare journal*, *6*(2), 94.	- The paper discusses the different types of AI relevant to healthcare and the specific processes and tasks they support.<br>- AI can perform healthcare tasks as well or better than humans, such as diagnosing diseases and guiding researchers in clinical trials.<br>- Implementation factors will prevent large-scale automation of healthcare professional jobs for a considerable period.<br>- Ethical issues related to the application of AI in healthcare are also discussed in the paper.
9	Guo, Y., Hao, Z., Zhao, S., Gong, J., & Yang, F. (2020). Artificial intelligence in health care: bibliometric analysis. *Journal of Medical Internet Research*, *22*(7), e18228.	- The paper provides a dynamic and longitudinal bibliometric analysis of health care-related AI publications.<br>- It reveals the growth rate of publications in health care-related AI research, which significantly increased from 2014 to 2019.<br>- It highlights the major health problems studied in AI research, including cancer, depression, Alzheimer's disease, heart failure, and diabetes.<br>- It shows the AI techniques with the highest impact on health care, such as artificial neural networks, support vector machines, and convolutional neural networks.<br>- It identifies the research hotspots in AI-related health care research, including nucleosides, convolutional neural networks, and tumor markers.<br>- It suggests that future AI research should focus on bridging the gap between AI health care research and clinical applications.
10	Hajkowicz, S., Sanderson, C., Karimi, S., Bratanova, A., & Naughtin, C. (2023). Artificial intelligence adoption in the physical sciences, natural sciences, life sciences, social sciences and the arts and humanities: A bibliometric analysis of research publications from 1960-2021. *Technology in Society*, *74*, 102260.	- The paper provides a comprehensive analysis of the worldwide adoption of AI technology across different fields of research from 1960 to 2021, using bibliometric analysis with 137 million peer-reviewed publications captured in the Lens.org database.<br>- It defines AI using a list of 214 phrases developed by expert working groups at the Organisation for Economic Cooperation and Development (OECD).<br>- The research findings reveal a surge in AI adoption across practically all research fields, including physical science, natural science, life science, social science, and the arts and humanities, in recent years.

		- The diffusion of AI beyond computer science was early, rapid, and widespread, with over half of all research fields being related to AI by 1972 and over 98% in current times.<br>- The paper notes that the current surge in AI adoption appears different from previous boom-bust cycles, suggesting that interdisciplinary AI application is likely to be sustained.
11	Klinger, J., Mateos-Garcia, J., & Stathoulopoulos, K. (2018). Deep learning, deep change? Mapping the development of the Artificial Intelligence General Purpose Technology. *arXiv preprint arXiv:1808.06355*.	- The paper analyses Deep Learning (DL) as a General Purpose Technology (GPT) and its rapid growth, diffusion into new fields, and impact in those fields, describing the changes in the geography of DL, including China's rise in AI rankings and the relative decline of several European countries.<br>- It identifies the consolidation of DL research hubs, suggesting a closing window of opportunity for new entrants.<br>- It studies of the regional drivers of DL clustering, highlighting the importance of proximity between GPT developers and adopters for collaboration and knowledge sharing.<br>- Discovery of a Chinese comparative advantage in DL after controlling for other factors, emphasizing the significance of data access and supportive policies for successful DL development.<br>- It addresses the gap in the literature regarding the links between disruptive GPT-like innovations and the rest of the economy.
12	Leite, M. L., de Loiola Costa, L. S., Cunha, V. A., Kreniski, V., de Oliveira Braga Filho, M., da Cunha, N. B., & Costa, F. F. (2021). Artificial intelligence and the future of life sciences. *Drug Discovery Today*, *26*(11), 2515-2526.	- The paper discusses how AI is revolutionizing the life sciences sector, particularly in biomedicine and healthcare, by improving disease diagnosis and patient outcomes, as well as reducing healthcare costs.<br>- It highlights the increasing use of AI in clinical trials, where patient information can be collected in real-time and explored using various AI tools.<br>- It mentions the use of mobile technologies associated with AI to improve aspects of disease diagnosis and treatment, offering patient-centric solutions.<br>- AI is shown to have applications in drug development and repurposing, enabling faster diagnosis, more efficient treatment, and the identification of data-driven hypotheses for scientists.<br>- The authors discuss the potential of big data analytics in the life sciences, emphasizing the importance of computational resources to handle the increasing volume and complexity of data.
13	Lundvall, B. Å., & Rikap, C. (2022). China's catching-up in artificial intelligence seen as a co-evolution of corporate and national innovation systems. *Research Policy*, *51*(1), 104395.	- The paper explores China's emergence as a lead country in artificial intelligence, highlighting the co-evolution of corporate and national innovation systems (NIS).<br>- It introduces the concept of "corporate innovation system" (CIS) and emphasizes the increasing importance of big companies as network leaders.<br>- It discusses the interaction within China's national innovation system and its openness, using Japan as a reference.<br>- It focuses on two Chinese tech giants, Alibaba and Tencent, and their innovation activities, which rely on knowledge sources within China's NIS and privileged access to Chinese data, also highlighting their international activities.

14	Noorbakhsh-Sabet, N., Zand, R., Zhang, Y., & Abedi, V. (2019). Artificial intelligence transforms the future of health care. *The American journal of medicine*, *132*(7), 795-801.	- The study looks at Artificial Intelligence (AI) in healthcare and its potential to unlock novel insights and accelerate breakthroughs by analyzing large, integrated datasets.<br>- Machine learning applications in healthcare have been used in clinical, translational, and public health settings, with a focus on privacy, data-sharing, and genetic information.<br>- Unsupervised learning, which aims to identify hidden patterns in data, has been used to explore data and generate novel hypotheses in various fields, including infectious disease distribution and heart failure research.<br>- Regularized logistic regression is an important tool for analyzing large-scale genotype or phenotype data, and sharing data across institutions is crucial for the success of genetic and biomedical studies.<br>- The development of complex algorithms in precision medicine offers compelling opportunities but also computational challenges, such as handling large volumes of data and integrating different data formats.
15	Radanliev, P., De Roure, D., Maple, C., & Santos, O. (2022). Forecasts on future evolution of artificial intelligence and intelligent systems. *IEEE Access*, *10*, 45280-45288.	- The study conducts a statistical analysis of research data records on artificial intelligence by year, country, language, and organization.<br>- It finds that the USA is the leading country in the field of artificial intelligence on a national level.<br>- Identifying English as the dominant language for disseminating results in the field of artificial intelligence.<br>- It points the confusion regarding the leading organization in the field of artificial intelligence, with conflicting results between the Chinese Academy of Sciences and the University of California.
16	Schwalbe, N., & Wahl, B. (2020). Artificial intelligence and the future of global health. *The Lancet*, *395*(10236), 1579-1586.	- The paper discusses the potential of artificial intelligence (AI) in addressing unique challenges in global health and accelerating the achievement of health-related sustainable development goals in low and middle-income countries (LMICs).<br>- It highlights the deployment of AI in LMICs for various health issues, particularly communicable diseases like tuberculosis and malaria, using machine learning and signal processing methods.<br>- It categorizes AI-driven health interventions into four areas: diagnosis, patient morbidity or mortality risk assessment, disease outbreak prediction and surveillance, and health policy and planning.<br>- It emphasizes the need for ethical, regulatory, and practical considerations in the development, testing, and widespread use of AI-driven interventions in global health.<br>- It suggests that despite being a nascent field, AI-driven health interventions have the potential to improve health outcomes in LMICs, but guidelines and a user-driven research agenda are necessary for equitable and ethical use.
17	Secinaro, S., Calandra, D., Secinaro, A., Muthurangu, V., & Biancone, P. (2021). The role	- The study analyses 288 peer-reviewed papers from Scopus, highlighting that the literature on AI in healthcare is emerging.

	of artificial intelligence in healthcare: a structured literature review. *BMC medical informatics and decision making*, *21*, 1-23.	- The main focus areas of AI in healthcare include health services management, predictive medicine, patient data and diagnostics, and clinical decision-making.<br>- The United States, China, and the United Kingdom contributed the highest number of studies in this field.<br>- AI has several applications in health services, such as supporting physicians in making diagnoses, predicting disease spread, and customizing treatment paths.<br>- The analysis of journals in this field confirms that AI in healthcare is an interdisciplinary research field, with contributions from medical journals or journals focused on technological growth in healthcare.
18	Simon, J. P. (2019). Artificial intelligence: scope, players, markets and geography. *Digital Policy, Regulation and Governance*, *21*(3), 208-237.	- The paper provides a comprehensive overview of the major trends in the field of artificial intelligence (AI).<br>- It identifies pioneering companies and the geographical distribution of AI companies, The USA dominates investment in AI, with significant growth and size in the region. Asia, North America, and Europe also show growth in investment, number of AI companies, and number of patents in the AI field. Europe has start-up hubs in Paris, London, and Berlin that focus intensively on AI.<br>- It notes the lack of consensus on a definition for the umbrella term of AI, and it highlights changes and advances in the past 60 years, together with the uncertainty in the demand for AI and the challenges in assessing the scope of disruptions and technological innovation associated with AI.<br>- The paper acknowledges the limitations of available research on economic and social aspects of AI, as most of the data come from consultancies or government publications, which may introduce some bias.
19	Tran, B. X., Vu, G. T., Ha, G. H., Vuong, Q. H., Ho, M. T., Vuong, T. T., ... & Ho, R. C. (2019). Global evolution of research in artificial intelligence in health and medicine: a bibliometric study. *Journal of clinical medicine*, *8*(3), 360.	- The study provides a global and historical overview of research on Artificial Intelligence (AI) in health and medicine, analyzing the publication volume, authors, and countries collaboration in the field of AI in health and medicine.<br>- It identifies major techniques used in AI research, including Robotic, Machine learning, Artificial neural network, Artificial intelligence, and Natural language process.<br>- It highlights the most frequent applications of AI in Clinical Prediction and Treatment.<br>- It identifies the highest number of cancer-related publications, followed by Heart Diseases and Stroke, Vision impairment, Alzheimer's, and Depression.<br>- It suggests future directions in AI research by pointing out the shortage of research on AI application to some high burden diseases and recommends the development of global and national protocols and regulations for the justification and adaptation of medical AI products.
20	Van Roy, V., Vertesy, D., & Damioli, G. (2019). *AI and robotics innovation: A sectoral and*	- The study finds a tremendous increase in AI patenting activities since 2013, with a significant boom in 2015-2016.

	geographical mapping using patent data (No. 433). GLO Discussion Paper.	- Concentration of AI patenting activities in the sectors of software programming and manufacturing of electronic equipment and machinery, and signs of cross-fertilization towards non-tech sectors.<br>- Mainland China leads in AI patenting activities, filing more patent families than Japan, its closest follower. The performance of China is particularly striking, with a significant increase in AI patents in recent years. The EU has a lower average number of patent families compared to China, South Korea, and the US.<br>- The US and South Korea have a comparative advantage in information and communication sectors, as well as manufacturing of electronic equipment. China and Japan have relative strength in manufacturing of electronic equipment and machinery.
21	Wolff, J., Pauling, J., Keck, A., & Baumbach, J. (2020). The economic impact of artificial intelligence in health care: systematic review. *Journal of medical Internet research*, *22*(2), e16866.	- The paper systematically reviews and summarizes cost-effectiveness studies dedicated to AI in healthcare.<br>- It identifies methodological deficits in existing economic impact assessments of AI in healthcare.<br>- It emphasizes the need for more comprehensive economic analyses in future studies to enable economic decisions regarding the implementation of AI technology in healthcare.<br>- It highlights the importance of considering initial investment and operational costs for AI infrastructure and services in economic impact assessments.<br>- It also suggests evaluating alternatives to achieve similar impact to provide a comprehensive comparison in economic impact assessments of AI in healthcare.
22	Xin, Y., Man, W., & Yi, Z. (2021). The development trend of artificial intelligence in medical: A patentometric analysis. *Artificial Intelligence in the Life Sciences*, *1*, 100006.	- The study uses Social Network Analysis (SNA) to characterize patent applications and cooperative networks in the AI-medical field.<br>- It maps a holistic landscape related to the AI-medical field using the Derwent Innovation Index database as the patent data source, identifying the United States as the foremost country developing related technologies and the primary target of patent filing by non-residents.<br>- It identifies hotspots in the AI-medical field, including medical image recognition, computer-aided diagnosis, disease monitoring, disease prediction, bioinformatics, and drug development.<br>- It finds that companies and academic institutions are the most active innovation subjects in the AI-medical field, with domestic collaboration being the major collaborative pattern.
23	Xu, D., Liu, B., Wang, J., & Zhang, Z. (2022). Bibliometric analysis of artificial intelligence for biotechnology and applied microbiology: Exploring research hotspots and frontiers. *Frontiers in Bioengineering and Biotechnology*, *10*, 998298.	- The paper provided a bibliometric framework to track biotechnological developments and explore specific knowledge areas.<br>- It analyzed 3,529 scientific papers on AI applications in biotechnology and applied microbiology published between 2000 and 2021. The United States had the highest number of publications, and 128 countries contributed to the research in this field.<br>- A total of 584 global institutions were involved in publishing these papers, with the Chinese Academy of Science being the most prolific.

		- The reference clusters from the studies were categorized into ten main headings, including deep learning, prediction, support vector machines (SVM), object detection, feature representation, synthetic biology, amyloid, human microRNA precursors, systems biology, and single-cell RNA-Sequencing.<br>- The research frontier keywords identified were microRNA (2012-2020) and protein-protein interactions (PPIs) (2012-2020).
24	Zhang, B., & Wang, H. (2021). Network proximity evolution of open innovation diffusion: A case of artificial intelligence for healthcare. *Journal of Open Innovation: Technology, Market, and Complexity*, *7*(4), 222.	- The paper constructs a theoretical framework that includes four proximity dimensions (technological, organizational, temporal, and network) to explore the key driving factors of open innovation diffusion in the context of a global industrial chain.<br>- It uses artificial intelligence for healthcare as an example to explore the key driving factors of open innovation diffusion in the context of a global industrial chain.<br>- Artificial intelligence technologies, such as machine learning and natural language processing, can be used to analyze large amounts of medical data, identify patterns, and make predictions, leading to more accurate diagnoses and personalized treatment plans.<br>- The empirical analysis verifies that technological proximity plays the leading role in innovation diffusion, while organizational and temporal proximities play secondary roles.<br>- The paper highlights the significance of open innovation diffusion in the patent system and emphasizes the potential support from the complex innovation network. It aims to guide policymakers in optimizing innovation management and policy implementation by understanding the evolution route of open innovation.
25	Zhang, D., Mishra, S., Brynjolfsson, E., Etchemendy, J., Ganguli, D., Grosz, B., ... & Perrault, R. (2021). The AI index 2021 annual report. *arXiv preprint arXiv:2103.06312*.	- The AI Index Report provides policymakers, researchers, executives, journalists, and the general public with unbiased and globally sourced data on artificial intelligence (AI) to develop a better understanding of the field.<br>- The report offers insights into the effects of COVID-19 on AI development, including the use of machine-learning-based techniques for COVID-related drug discovery and the impact on AI hiring and private investment.<br>- The shift to virtual formats for AI research conferences due to the pandemic may have led to increased participation, as evidenced by significant spikes in attendance.<br>- The report also includes information on jobs in AI, datasets, and computational experiments, offering practical resources for researchers and practitioners in the field.

S2 Number of AI-Related Life Science Articles by Country (2000-2022)

Lead author country	Total Numbers		
	All articles	PubMed	Conference Proceedings
Afghanistan	15	14	1
Albania	7	6	1
Algeria	217	177	40
Antigua and Barbuda	4	4	0
Argentina	734	720	14
Armenia	22	21	1
Australia	9,113	8,709	404
Austria	2,230	2,157	73
Azerbaijan	24	22	2
Bahamas	1	1	0
Bahrain	41	18	23
Bangladesh	732	460	272
Barbados	4	3	1
Belarus	36	28	8
Belgium	3,360	3,232	128
Belize	2	2	0
Benin	12	11	1
Bermuda	1	1	0
Bhutan	6	6	0
Bolivia	4	4	0
Bosnia and Herzegovina	44	31	13
Botswana	7	3	4
Brazil	5,435	5,215	220
Brunei	36	29	7
Bulgaria	211	176	35
Burkina Faso	7	7	0
Burundi	18	17	1
Cambodia	19	16	3
Cameroon	43	43	0
Canada	12,578	11,976	602
Cayman Islands	3	3	0
Chile	526	506	20
China	73,129	69,684	3,445
Colombia	657	631	26
Congo	4	3	1
Costa Rica	43	40	3
Croatia	367	332	35
Cuba	114	106	8
Cyprus	220	189	31
Czech Republic	1,006	950	56
Democratic Republic of the Congo	2	2	0
Denmark	2,352	2,270	82
Dominica	1	1	0
Dominican Republic	2	2	0
East Timor	1	0	
Ecuador	107	85	22
Egypt	1,115	1,013	102

Lead author country	Total Numbers		
	All articles	PubMed	Conference Proceedings
El Salvador	1	0	1
Eritrea	1	1	0
Estonia	175	167	8
Eswatini	5	5	0
Ethiopia	276	274	2
Faroe Islands	1	1	0
Fiji	29	22	7
Finland	1,852	1,769	83
France	10,121	9,793	328
French Guiana	5	5	0
French Polynesia	6	6	0
Gabon	2	2	0
Gambia	7	6	1
Georgia	26	26	0
Germany	18,759	18,037	722
Ghana	127	120	7
Greece	2,126	2,028	98
Greenland	3	2	1
Grenada	4	4	0
Guadeloupe	8	8	0
Guatemala	6	5	1
Guinea	2	2	0
Haiti	1	1	0
Holy See	1	1	0
Honduras	3	0	3
Hong Kong	1,483	1,423	60
Hungary	868	838	30
Iceland	75	71	4
India	12,560	9,171	3,389
Indonesia	991	239	752
Iran	4,645	4,533	112
Iraq	208	122	86
Ireland	1,318	1,248	70
Isle of Man	1	1	0
Israel	2,610	2,509	101
Italy	12,134	11,675	459
Ivory Coast	6	6	0
Jamaica	3	3	0
Japan	15,263	13,859	1,404
Jordan	252	239	13
Kazakhstan	103	72	31
Kenya	107	105	2
Kosovo	2	2	0
Kuwait	65	64	1
Kyrgyzstan	4	4	0
Latvia	52	45	7
Lebanon	128	121	7
Lesotho	1	1	0
Liberia	1	0	1
Libya	27	14	13
Liechtenstein	3	1	2
Lithuania	213	200	13

Lead author country	Total Numbers		
	All articles	PubMed	Conference Proceedings
Luxembourg	170	155	15
Macao	363	354	9
Macedonia	48	40	8
Madagascar	3	3	0
Malawi	12	10	2
Malaysia	1,875	1,594	281
Maldives	2	1	1
Mali	9	9	0
Malta	53	41	12
Martinique	4	4	0
Mauritius	16	8	8
Mexico	1,673	1,587	86
Micronesia	1	1	0
Moldova	6	3	3
Monaco	8	8	0
Mongolia	11	9	2
Montenegro	10	9	1
Morocco	279	238	41
Mozambique	3	3	0
Myanmar	12	10	2
Nepal	67	51	16
Netherlands	7,253	6,992	261
New Caledonia	8	8	0
New Zealand	1,245	1,165	80
Niger	3	3	0
Nigeria	269	244	25
Norway	1,493	1,429	64
Oman	66	49	17
Pakistan	1,269	1,148	121
Palestine	24	23	1
Panama	23	18	5
Papua New Guinea	5	5	0
Paraguay	15	9	6
Peru	126	83	43
Philippines	251	114	137
Poland	3,117	2,996	121
Portugal	2,087	1,925	162
Puerto Rico	57	55	2
Qatar	318	295	23
Réunion	5	5	0
Romania	908	728	180
Russia	2,050	1,721	329
Rwanda	10	9	1
Saint Kitts and Nevis	2	2	0
Saint Vincent and the Grenadines	1	1	0
Sao Tome and Principe	3	3	0
Saudi Arabia	1,866	1,769	97
Senegal	11	9	2
Serbia	554	533	21
Sierra Leone	1	1	0
Singapore	3,194	3,001	193

Lead author country	Total Numbers		
	All articles	PubMed	Conference Proceedings
Slovakia	239	221	18
Slovenia	626	607	19
Somalia	2	2	0
South Africa	845	800	45
South Korea	12,264	11,794	470
South Sudan	12	12	0
Spain	9,212	8,891	321
Sri Lanka	126	58	68
Sudan	18	12	6
Sweden	3,434	3,282	152
Switzerland	4,977	4,781	196
Syria	20	18	2
Taiwan	5,767	5,486	281
Tajikistan	6	5	1
Tanzania	29	29	0
Thailand	1,023	899	124
Togo	2	2	0
Tonga	1	1	0
Trinidad and Tobago	18	17	1
Tunisia	271	238	33
Turkey	3,146	2,881	265
Uganda	38	35	3
Ukraine	197	150	47
United Arab Emirates	489	389	100
United Kingdom	21,215	20,278	937
United States	101,195	96,775	4,420
Uruguay	79	76	3
Uzbekistan	17	6	11
Vanuatu	1	1	0
Venezuela	66	62	4
Vietnam	432	389	43
Virgin Islands (British)	8	1	7
Virgin Islands (U.S.)	1	1	0
Yemen	24	20	4
Zambia	8	8	0
Zimbabwe	18	16	2

S3 Explanation of Concepts recorded in OpenAlex

The OpenAlex database tags articles with multiple concepts representing their topical focus using a state-of-the art machine learning classifier based on titles and abstracts, with confidence scores indicating relevance.[1] These scientific concepts are organized hierarchically, with 19 root-level concepts branching into six levels of specific topics. When a lower-level concept is mapped, all of its parent concepts are mapped as well, ensuring comprehensive coverage. This structure supports a rich network of interconnected scientific entities, facilitating advanced querying and analysis.[2] After performing a review of pertinent literature, Wang et al 2020 (p. 399) state, for example: “Numerous studies seem to confirm that machine curated results in MAG achieve reasonable if not greater accuracy over commercial data sets with considerable amount of human effort.” We use these concepts for two different purposes. First, we use the level 0 concepts to identify life science relevant conference proceedings publications. Second, we use the level 1 concepts to create heatmaps depicting the life science content of articles in our sample. A list of all OpenAlex concepts and their tree position can be found here:

https://docs.google.com/spreadsheets/d/1LBFHjPt4rj_9r0t0TTAlT68NwOtNH8Z21lBMsJDMoZg/edit#gid=575855905.

S4 List of A* ranked Conference Proceedings

2009 IEEE Computer Society Conference on Computer Vision and Pattern Recognition Workshops; 2009 IEEE Conference on Computer Vision and Pattern Recognition; 2020 IEEE Conference on Virtual Reality and 3D User Interfaces (VR); 2020 IEEE Conference on Virtual Reality and 3D User Interfaces Abstracts and Workshops (VRW); 2021 36th IEEE/ACM International Conference on Automated Software Engineering (ASE); 2021 IEEE Conference on Virtual Reality and 3D User Interfaces Abstracts and Workshops (VRW); 2021 IEEE International Conference on Data Mining (ICDM); 2021 IEEE/CVF International Conference on Computer Vision (ICCV); 2021 International Conference on Data Mining Workshops (ICDMW); 2022 10th International Conference on Reliability, Infocom Technologies and Optimization (Trends and Future Directions) (ICRITO); 2022 IEEE 38th International Conference on Data Engineering (ICDE); 2022 IEEE 63rd Annual Symposium on Foundations of Computer Science (FOCS); 2022 IEEE Conference on Virtual Reality and 3D User Interfaces (VR); 2022 IEEE Conference on Virtual Reality and 3D User Interfaces Abstracts and Workshops (VRW); 2022 IEEE International Conference on Data Mining (ICDM); 2022 IEEE International Conference on Data Mining Workshops (ICDMW); 2022 IEEE International Conference on Pervasive Computing and Communications Workshops and other Affiliated Events (PerCom Workshops); 2022 IEEE International Symposium on Mixed and Augmented Reality Adjunct (ISMAR-Adjunct); 2022 IEEE/CVF Conference on Computer Vision and Pattern Recognition (CVPR); 2022 IEEE/CVF Conference on Computer Vision and Pattern Recognition Workshops (CVPRW); 2022 International Conference on Robotics and Automation (ICRA); AAAI Spring Symposium Combining Machine Learning with Knowledge Engineering; ACM Multimedia; CHI '00 extended abstracts on Human factors in computer systems - CHI '00; CHI '03 extended abstracts on Human factors in computer systems - CHI '03; CHI Conference on Human Factors in Computing Systems; CHI Conference on Human Factors in Computing Systems Extended Abstracts; Computer and Communications Security; Computer Vision and Pattern Recognition; Conference of the European Chapter of the Association for Computational Linguistics; Conference on Learning Theory; Conference on Management of Data; Empirical Methods in Natural Language Processing; European Conference on Computer Vision; Findings of the Association for Computational Linguistics: ACL 2022; Findings of the Association for Computational Linguistics: NAACL 2022; Human Factors in Computing Systems; IEEE INFOCOM 2022 - IEEE Conference on Computer Communications; IEEE INFOCOM 2022 - IEEE Conference on Computer Communications Workshops (INFOCOM WKSHPS); IEEE Symposium on Security and Privacy; Information Processing in Sensor Networks; International ACM SIGIR Conference on Research and Development in Information Retrieval; International Conference on Automated Planning and Scheduling; International Conference on Computer Graphics and Interactive Techniques; International Conference on Computer Vision; International Conference on Data Mining; International Conference on Learning Representations; International Conference on Machine Learning; International Conference on Management of Data; International Conference on Neural Information Processing; International Conference on Pervasive Computing; International Conference on Robotics and Automation; International Conference on Software Engineering; International Joint Conference on Artificial Intelligence; Knowledge Discovery and Data Mining; Meeting of the Association for Computational Linguistics; Neural Information Processing Systems; North American Chapter of the Association for Computational Linguistics; Principles of Knowledge Representation and Reasoning; Proceedings of the 2021 Conference on Empirical Methods in Natural Language Processing; Proceedings of the 2022 ACM on Asia Conference on Computer and Communications Security; Proceedings of the 2022 ACM SIGSAC Conference on Computer and Communications Security; Proceedings of the 2022 Conference of the North American Chapter of the Association for Computational Linguistics: Human Language Technologies; Proceedings of the 2022 International Conference on Management of Data; Proceedings of the 23rd ACM Conference on Economics and Computation; Proceedings of the 28th ACM SIGKDD Conference on Knowledge Discovery and Data Mining; Proceedings of the 30th ACM International Conference on Multimedia; Proceedings of the 30th ACM Joint European Software Engineering Conference and Symposium on the Foundations of Software

Engineering; Proceedings of the 44th International Conference on Software Engineering; Proceedings of the 45th International ACM SIGIR Conference on Research and Development in Information Retrieval; Proceedings of the 60th Annual Meeting of the Association for Computational Linguistics (Volume 1: Long Papers); Proceedings of the ninth ACM international conference on Multimedia - MULTIMEDIA '01; Proceedings of the second international joint conference on Autonomous agents and multiagent systems - AAMAS '03; Proceedings of the Thirty-First International Joint Conference on Artificial Intelligence; Symposium on the Theory of Computing; USENIX Security Symposium; Very Large Data Bases; World Conference on WWW and Internet

S5 List of AI-related Keywords used in Search Strategy

action recognition, human action recognition, activity recognition, human activity recognition, adaboost, adaptive boosting, adversarial network, generative adversarial network, ambient intelligence, ant colony, ant colony optimisation, artificial intelligence, human aware artificial intelligence, association rule, autoencoder, autonomic computing, autonomous vehicle, autonomous weapon, backpropagation, Bayesian learning, bayesian network, bee colony, artificial bee colony algorithm, blind signal separation, bootstrap aggregation, brain computer interface, brownboost, chatbot, classification tree, cluster analysis, cognitive automation, cognitive computing, cognitive insight system, cognitive modelling, collaborative filtering, collision avoidance, community detection, computational intelligence, computational pathology, computer vision, cyber physical system, data mining, decision tree, deep belief network, deep learning, dictionary learning, dimensionality reduction, dynamic time warping, emotion recognition, ensemble learning, evolutionary algorithm, differential evolution algorithm, multi-objective evolutionary algorithm, evolutionary computation, face recognition, facial expression recognition, factorisation machine, feature engineering, feature extraction, feature learning, feature selection, firefly algorithm, fuzzy c, fuzzy environment, fuzzy logic, fuzzy number, fuzzy set, intuitionistic fuzzy set, fuzzy system, t s fuzzy system, Takagi-Sugeno fuzzy systems, gaussian mixture model, gaussian process, genetic algorithm, genetic programming, gesture recognition, gradient boosting, gradient tree boosting, graphical model, gravitational search algorithm, hebbian learning, hierarchical clustering, high-dimensional data, high-dimensional feature, high-dimensional input, high-dimensional model, high-dimensional space, high-dimensional system, image classification, image processing, image recognition, image retrieval, image segmentation, independent component analysis, inductive monitoring, instance-based learning, intelligence augmentation, intelligent agent, intelligent software agent, intelligent classifier, intelligent geometric computing, intelligent infrastructure, Kernel learning, K-means, latent dirichlet allocation, latent semantic analysis, latent variable, layered control system, learning automata, link prediction, logitboost, long short term memory (LSTM), lpboost, machine intelligence, machine learning, extreme machine learning, machine translation, machine vision, madaboost, MapReduce, Markovian, hidden Markov model, memetic algorithm, meta learning, motion planning, multi task learning, multi-agent system, multi-label classification, multi-layer perceptron, multinomial naïve Bayes, multi-objective optimisation, naïve Bayes classifier, natural gradient, natural language generation, natural language processing, natural language understanding, nearest neighbour algorithm, neural network, artificial neural network, convolutional neural network, deep convolutional neural network, deep neural network, recurrent neural network, neural turing, neural turing machine, neuromorphic computing, non negative matrix factorisation, object detection, object recognition, obstacle avoidance, pattern recognition, pedestrian detection, policy gradient methods, Q-learning, random field, random forest, rankboost, recommender system, regression tree, reinforcement learning, relational learning, statistical relational learning, robot, biped robot, humanoid robot, human-robot interaction, industrial robot, legged robot, quadruped robot, service robot, social robot, wheeled mobile robot, rough set, rule learning, rule-based learning, self-organising map, self-organising structure, semantic web, semi-supervised learning, sensor fusion, sensor data fusion, multi-sensor fusion, sentiment analysis, similarity learning, simultaneous localisation mapping, single-linkage clustering, sparse representation, spectral clustering, speech recognition, speech to text, stacked generalisation, stochastic gradient, supervised learning, support vector regression, swarm intelligence, swarm optimisation, particle swarm optimisation, temporal difference learning, text mining, text to speech, topic model, totalboost, trajectory planning, trajectory tracking, transfer learning, trust region policy optimisation, unmanned aerial vehicle, unsupervised learning, variational inference, vector machine, support vector machine, virtual assistant, visual servoing, xgboost

List obtained from Baruffaldi et al. 2020[3]

S6 Sample Creation and Accuracy

To evaluate the accuracy with which the applied search approach allows us to identify AI focused life science research, we assess precision and comprehensiveness. First, we analyze precision, which is the extent to which the articles we retrieve are relevant, i.e., actually contain AI focused life science research. Second, we analyze comprehensiveness, which is the extent to which all relevant articles are retrieved with our search approach.

Precision

To evaluate precision, we took a random sample of 150 articles from our sample of PubMed publications and hired two independent raters to categorize the documents into having an AI application. Additionally, we took a random sample of 150 conference proceedings publications and asked the same two raters to categorize the documents as being linked to the life sciences. Across all 300 manually checked documents, the observed agreement among raters was 96%.

Out of the 150 PubMed publications, 135 (90%) were categorized as having some form of AI applications. Out of the 150 conference proceedings publications, 139 (93%) were categorized as being related to the life sciences. The AI relevance of conference proceedings publications and the life science relevance of PubMed publications is given by construction of our dataset.

Comprehensiveness

To assess comprehensiveness, we identified special issues on Artificial Intelligence published by journals indexed in PubMed. Specifically, we searched for editorials published in 2022 that contained the keywords "special issue" and "artificial intelligence," yielding 15 special issues with a total of 184 articles. In total, 170 out of 184 papers (92%) were included in our dataset.

Upon manual inspection of the 14 non-recalled articles, we find that five non-recalled articles contain AI-identifying keywords in the main text but not in the title and abstract, while two non-recalled articles use more general terms such as "algorithm", thereby evading our applied keyword identification. The remaining three articles are commentaries without a dedicated abstract, which reduces the likelihood of keyword-based recalls from titles alone. There is some variance in recall by special issue (with a minimum of about 79% at the issue level). Table S6 provides an overview of the special issues and the corresponding proportion of articles covered in our sample.

Special Issue	Journal	# Publications in Special Issue	# Publications in our sample	% in our sample
Artificial Intelligence and the Risk for Intuition Decline in Clinical Medicine	The American Journal of Gastroenterology	2	2	100%
Machine Learning in Orthopaedics: Venturing Into the Valley of Despair	Arthroscopy	2	2	100%
Transforming public health through artificial intelligence, machine learning and internet of things	Socio-Economic Planning Sciences	8	7	88%
Deep Learning and Machine Learning in Bioinformatics	International Journal of Molecular Sciences	22	19	86%
Artificial Intelligence in Image-Based Screening, Diagnostics, and Clinical Care of Cardiopulmonary Diseases	Diagnostics	14	12	86%
Imaging AI in Practice	Radiology: Artificial Intelligence	19	15	79%
Applications of Artificial Intelligence in Biomarker Research	Cancer Biomarkers	9	8	89%
Artificial intelligence in musculoskeletal radiology	Skeletal Radiology	21	21	100%
Veterinary Radiology & Ultrasound Special Issue on Artificial Intelligence	Veterinary Radiology & Ultrasound	12	11	92%
Prediction Models for COVID-19 Mortality Using Artificial Intelligence	Journal of Personalized Medicine	16	16	100%
Special Issue Artificial Intelligence in Oral Health	Diagnostics	15	14	93%
Special issue deep learning for multimedia healthcare	Multimedia Systems	11	11	100%
Special issue on machine learning and deep learning in magnetic resonance	NMR in Biomedicine	10	10	100%
Special Issue: Machine Learning for Computer-Aided Diagnosis in Biomedical Imaging	Diagnostics	16	16	100%
Special issue: Artificial intelligence in genomics	Human Genetics	7	6	86%
**Total**		**184**	**170**	**92%**

S6. Special issues and publications included in our sample.

We also compared the search approach with a second search strategy presented by Liu and colleagues.[4] We find that the search approach by Liu and colleagues, which relies on a smaller set of keywords to identify AI-related research, would yield a sample of approximately 180,000 AI-related life science publications in PubMed. Of these 180,000 articles, 93% are included in our sample obtained by applying the search strategy proposed by Baruffaldi and colleagues. In addition, a manual review of a random set of 100 PubMed articles identified by the Liu et al. yielded a precision of 94%. Although the approach proposed by Liu and colleagues is thus slightly more precise, we opt for the search strategy of Baruffaldi et al. for retrieving over twice as many relevant articles.

Overall, these numbers corroborate the accuracy and completeness of our sample of AI life science research.

S7 AI life science research in journals versus conference proceedings

Because AI life sciences research is conducted at the intersection of the biomedical and computer sciences, we sought to include publications from both domains in the creation of the global atlas depicting the research enterprise. The primary means of publication differ between the two domains. While the biomedical sciences usually communicate findings through journal publications, computer science disseminates new research primarily through the publication of conference proceedings. The inherent and multifaceted implications of these differences, for example, in terms of knowledge velocity, article size, and citation patterns, are beyond the scope of our manuscript. Nevertheless, for informational purposes, we offer split-sample analyses to complement our findings presented in the main manuscript.

Supplementary Figures S7.1 to S7.9 present the results of these split-sample analyses, with the respective figures (A) showing the results for journal publications indexed in PubMed and (B) showing the results for conference proceedings publications contained in OpenAlex.

In summary, we find that AI life science research published in both journals and conference proceedings has followed an exponential growth pattern in recent years (S7.1). The geography of production is also quite similar in biomedicine and computer science, with the exception that Asian countries, especially India, contribute more to conference proceedings publications (S7.2). Again, S2 shows the numerical statistics at the country level. Not surprisingly, there is a difference in the topical focus of biomedical sciences and computer science (S7.3). While the biomedical sciences have a stronger focus on health-related topics (S7.3A), the computer sciences place more emphasis on basic technological research, such as human-computer interaction, simulation or mathematical optimization (S7.3B). Similarly, publications in conference proceedings are less likely to deal with clinically applied research than biomedical research published in journals. However, the geographical focus on Asia as a producer of conference proceedings publications is also reflected in the clinical research subset (S7.4). Quality-adjusted productivity shows similar geographical patterns in biomedical and computer sciences, both led by the regions of North America and Europe (S7.5). Over time, Europe shows a consistent decline in its contributions to high-ranking outlets, however this decline seems stronger in the computational than the biomedical

sciences (S7.6). In terms of relevance of the research produced, the world regions of Northern America and Oceania generate a consistent citation premium over other world regions for their AI life science research (S7.7). European research, on the other hand, generates a citation premium only for journal publications. The higher productivity of Asian countries for conference proceedings publications is not associated with a citation premium. Finally, international collaborations seem to be less frequent for conference proceedings publications than for journal publications. Irrespective of the research field, international collaborations generate a citation premium compared to national collaborations, but have stagnated (S7.8 - S7.9).

S7.1A. Exponential Growth – PubMed

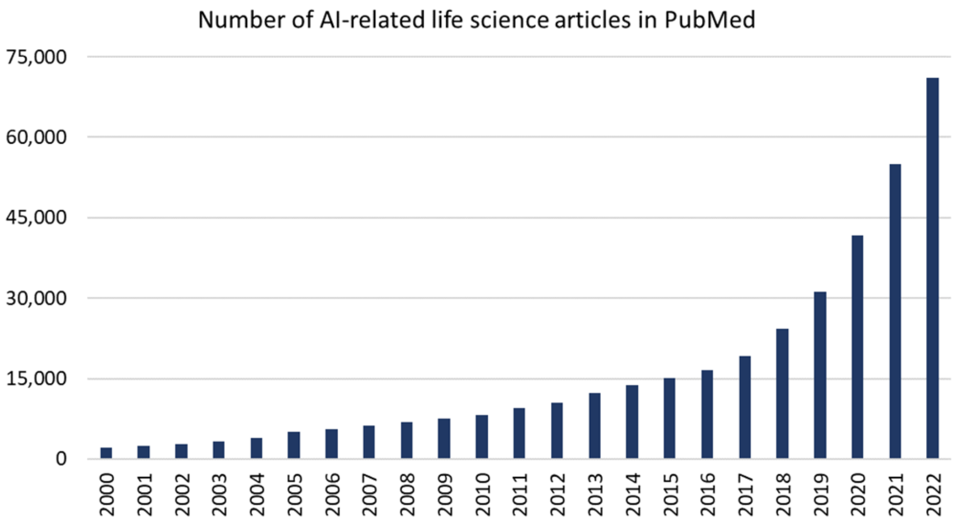

Fig S7.1A | Evolution of the AI research enterprise in the life sciences. Yearly counts of articles recorded in PubMed (n = 374,501) with AI-related keywords in titles or abstracts from 2000 to 2022.

S7.1B. Exponential Growth – Conference Proceedings

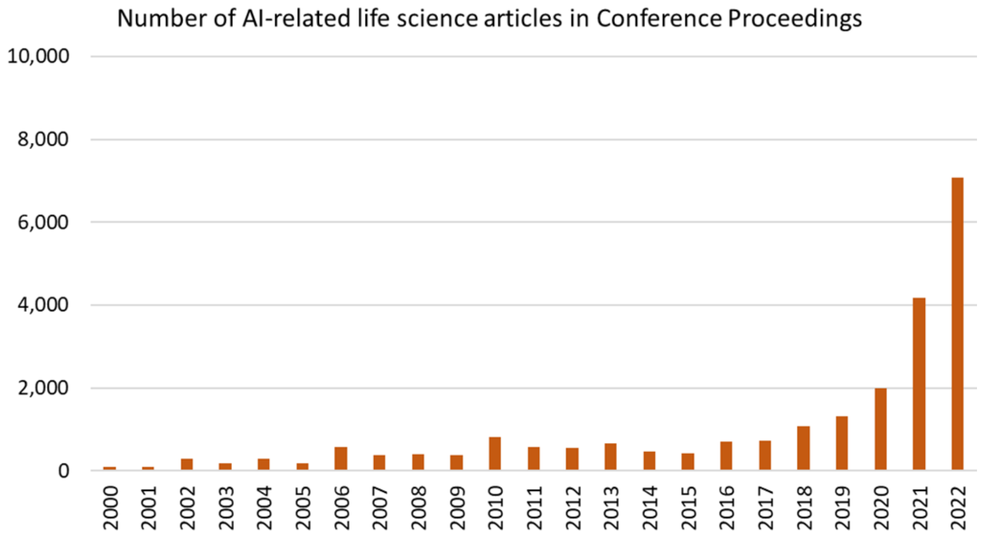

Fig S7.1B | Evolution of the AI research enterprise in the life sciences. Yearly counts of articles (n = 23,466) published in conference proceedings and recorded in OpenAlex with AI-related keywords in titles or abstracts from 2000 to 2022.

S7.2A. Productivity World Map - PubMed

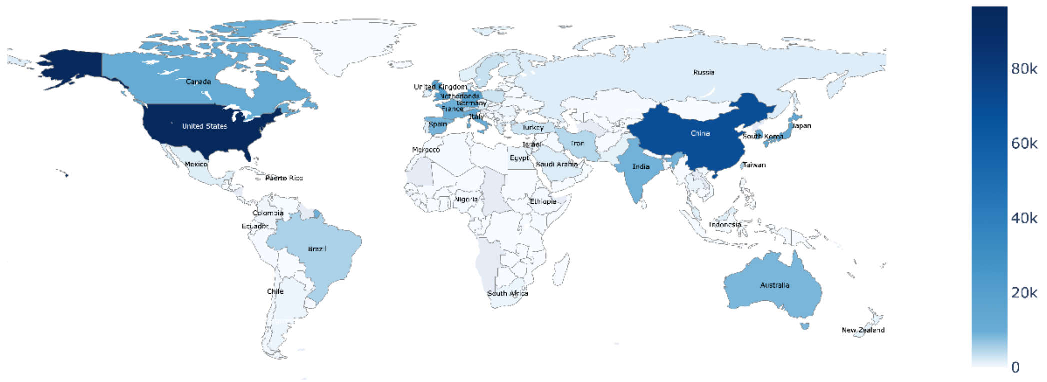

Fig S7.2A | Geography of the AI life science research enterprise in terms of productivity. Counts of AI-focused life science articles recorded in PubMed by country, cumulated for the years 2000 to 2022 (n = 374,501).

S7.2B. Productivity World Map – Conference Proceedings

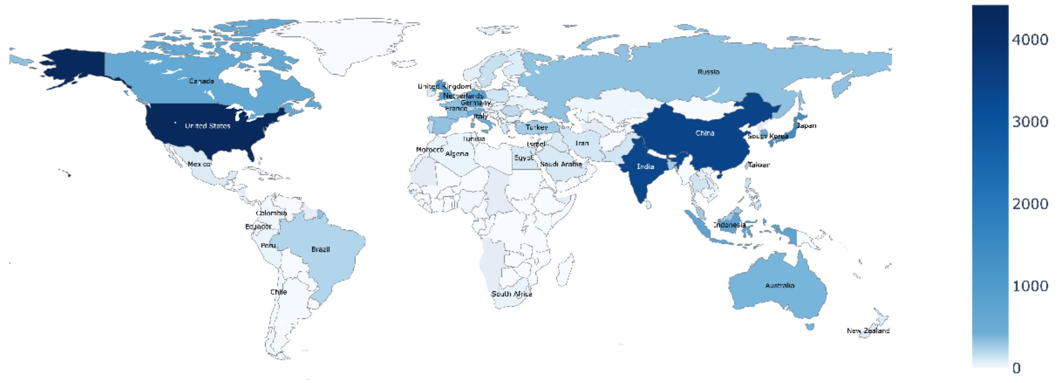

Fig S7.2B | Geography of the AI life science research enterprise in terms of productivity. Counts of AI-focused life science articles published in conference proceedings and recorded in OpenAlex by country, cumulated for the years 2000 to 2022 (n = 23,466).

S7.3A. Heatmap of content focus – PubMed

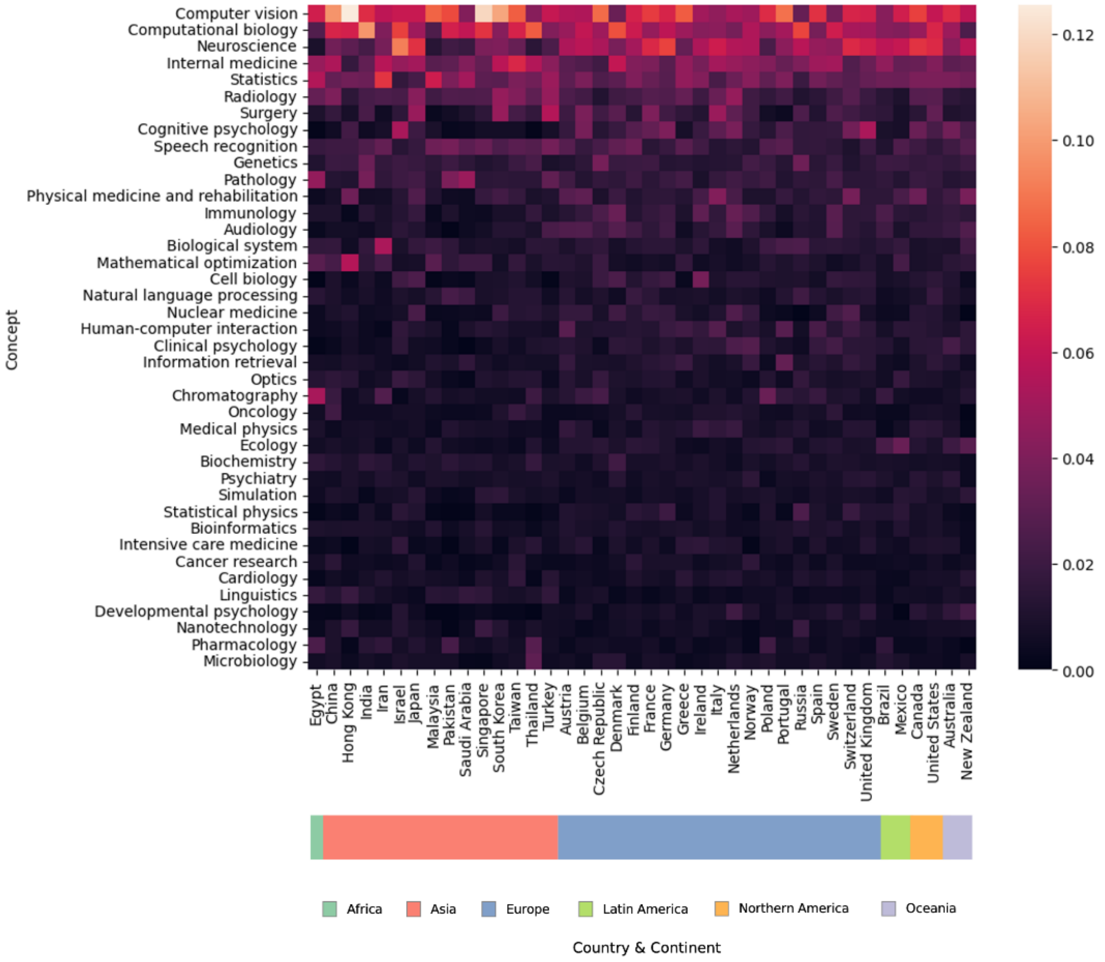

Fig S7.3A | Heatmap of relative country focus with respect to publication topics. The horizontal axis enlists the 40 most productive countries grouped by geographic region. The vertical axis depicts the underlying publication topics in descending order (computer vision being the most frequently researched topic). The color scheme of the heatmap reflects the percentage share of country-specific productivity according to the number of articles recorded in PubMed for a given publication topic (n = 253,615).

S7.3B. Heatmap of content focus – Conference Proceedings

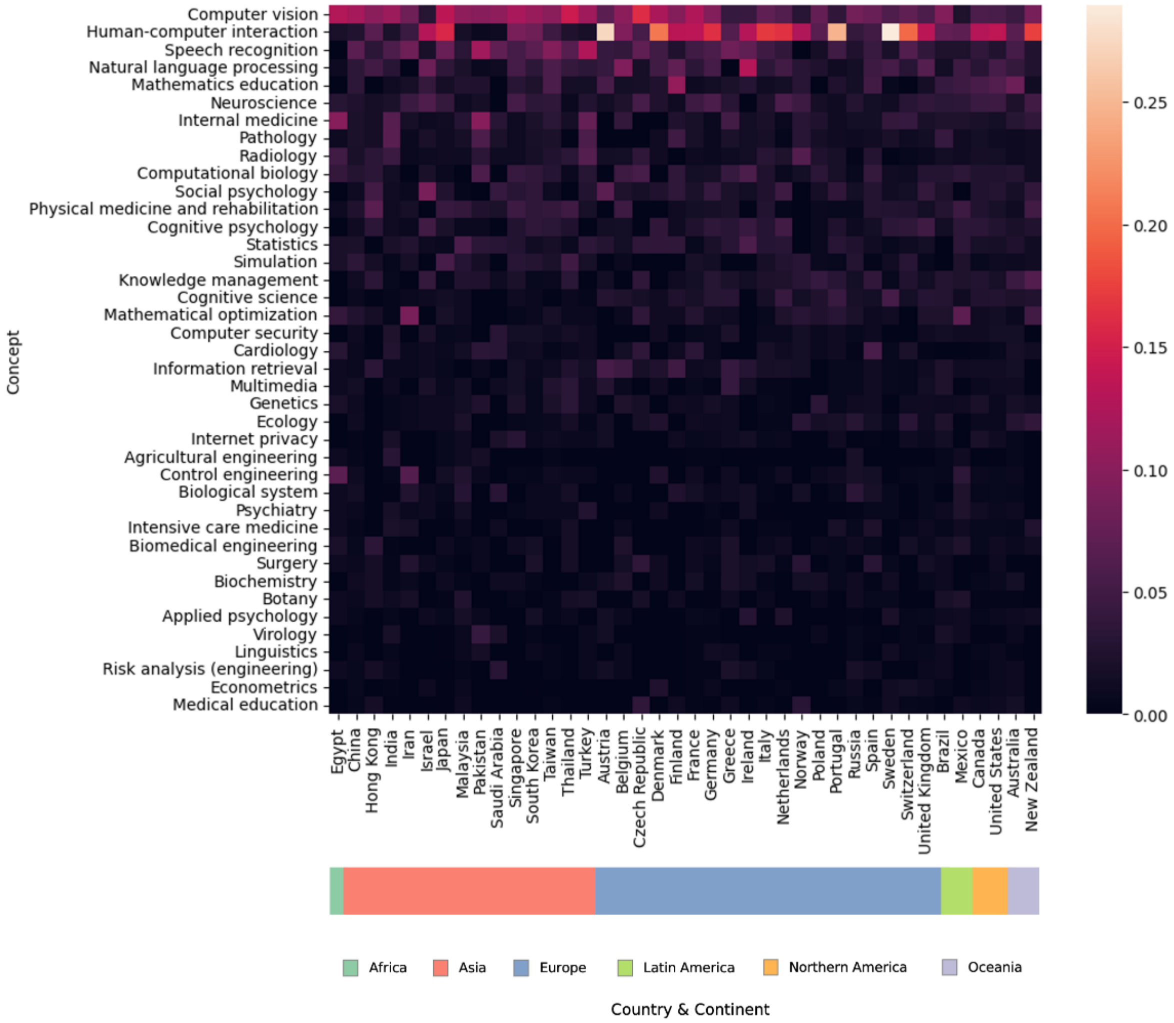

Fig S7.3A | Heatmap of relative country focus with respect to publication topics. The horizontal axis enlists the 40 most productive countries grouped by geographic region. The vertical axis depicts the underlying publication topics in descending order (computer vision being the most frequently researched topic). The color scheme of the heatmap reflects the percentage share of country-specific productivity according to the number of articles published in conference proceedings and recorded in OpenAlex for a given publication topic (n = 17,626).

S7.4A. Clinical Research – PubMed

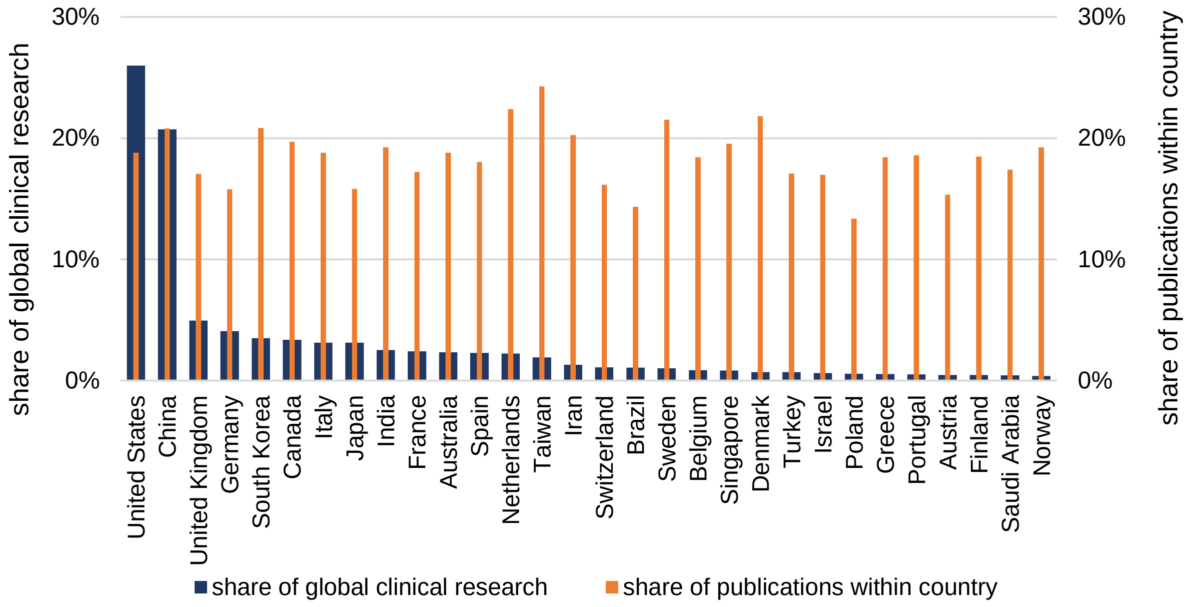

Fig S7.4A | Clinical AI research across countries. The share of a country's clinical research relative to global clinical research production (primary y-axis) and relative to all publications within the same country (secondary y-axis) for the 30 most productive countries in terms of clinical articles recorded in PubMed (n = 65,898).

S7.4B. Clinical Research – Conference Proceedings

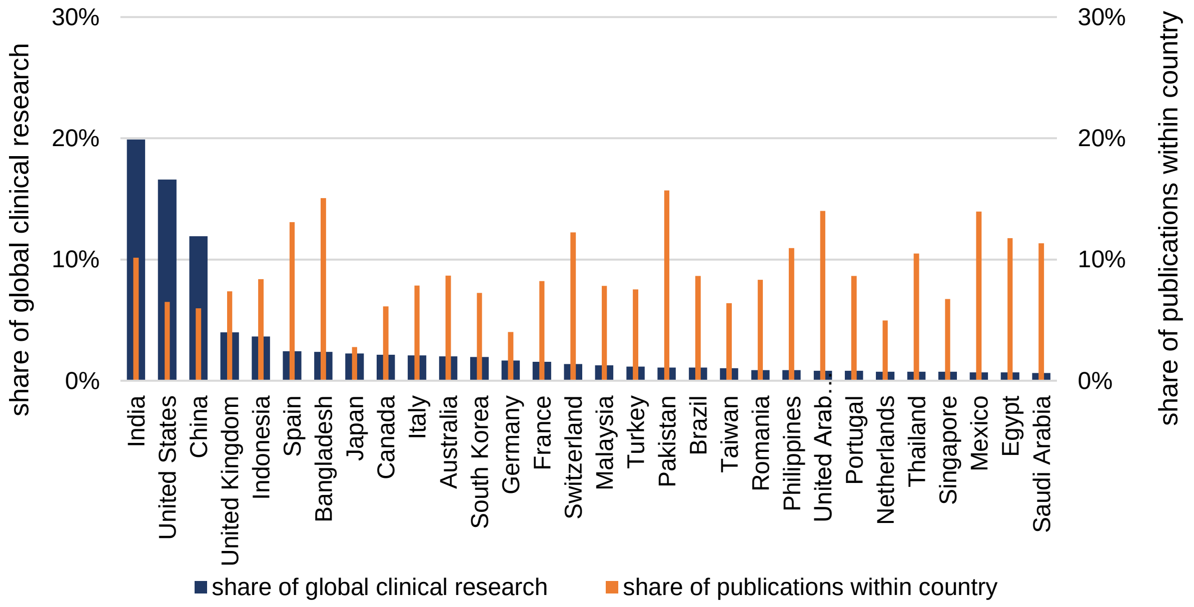

Fig S7.4B | Clinical AI research across countries. The share of a country's clinical research relative to global clinical research production (primary y-axis) and relative to all publications within the same country (secondary y-axis) for the 30 most productive countries in terms of clinical articles published in conference proceedings and recorded in OpenAlex (n =1,543).

S7.5A. Quality adjusted World Map – PubMed

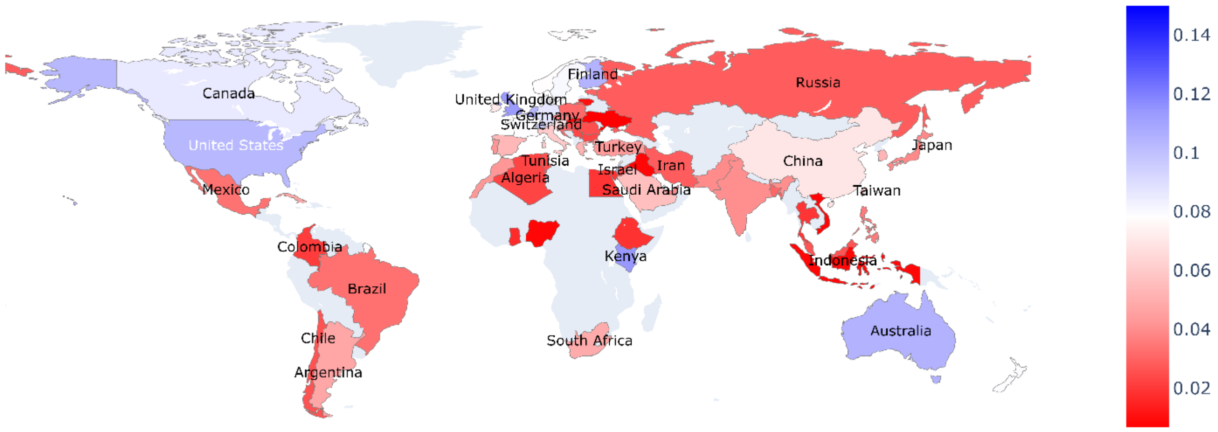

Fig S7.5A | Geography of the AI life science research enterprise in terms of quality-adjusted productivity. Percentage shares of AI-focused life science articles published in high-ranked outlets recorded in PubMed by country, cumulated for the years 2000 to 2022 (n = 372,822). The analysis is limited to countries with at least 100 publications.

S7.5B. Quality adjusted World Map – Conference Proceedings

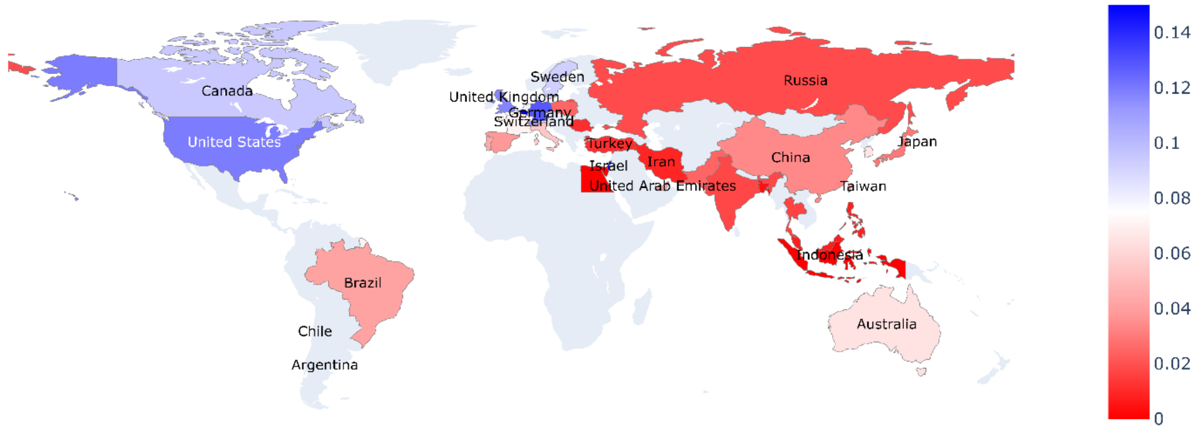

Fig S7.5B | Geography of the AI life science research enterprise in terms of quality-adjusted productivity. Percentage shares of AI-focused life science articles published in high-ranked conference proceedings recorded in OpenAlex by country, cumulated for the years 2000 to 2022 (n = 21,491). The analysis is limited to countries with at least 100 publications.

S7.6A. Average quality within region across time – PubMed

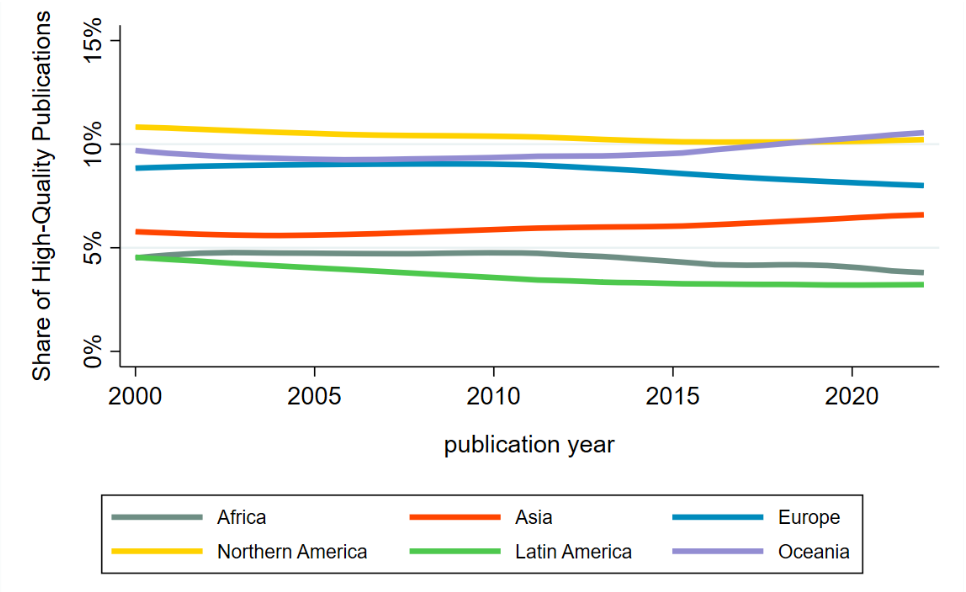

Fig6 | Geography of the AI life science research enterprise in terms of quality-adjusted productivity. Percentage shares of AI life science articles published in high-ranked outlets recorded in PubMed by geographic region and per year (n = 374,501).

S7.6B. Average quality within region across time – Conference Proceedings

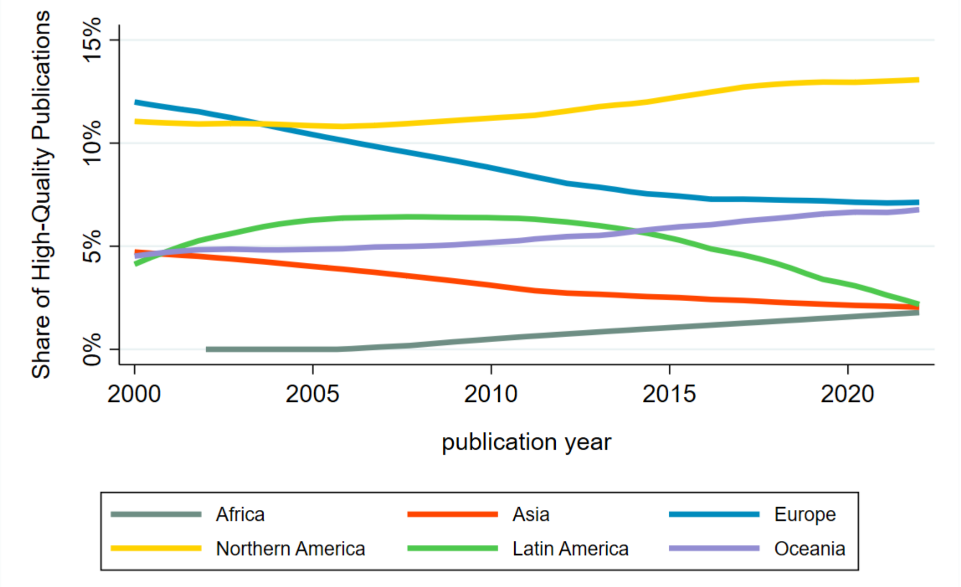

Fig6 | Geography of the AI life science research enterprise in terms of quality-adjusted productivity. Percentage shares of AI life science articles published in high- ranked conference proceedings recorded in OpenAlex by geographic region and per year (n = 23,466).

S7.7A. Relevance of AI research – PubMed

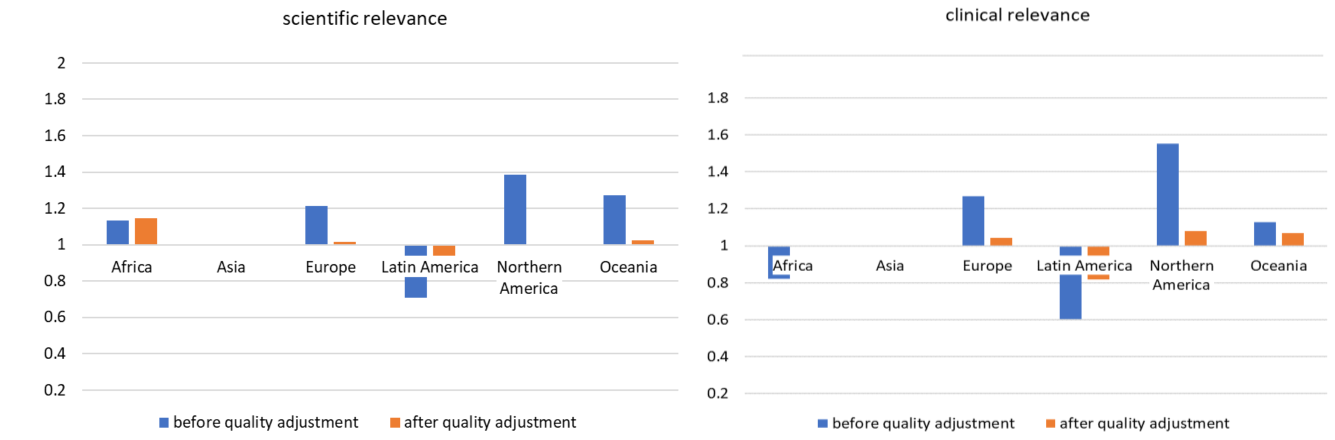

Fig S7.7A | Geography of the relevance of AI life science articles in terms of forward citations in life science research and clinical research. All bars depict incidence rate ratios (IRRs) obtained from negative binomial regressions of citations on dummy variables for the geography of articles recorded in PubMed (n = 374,501), with the most productive region, Asia, serving as base category. Blue bars show unadjusted estimates (only accounting for publication year), whereas orange bars also include controls for quality variation across publishing outlets. The left panel considers all citations and the right panel only citations by clinical research.

S7.7B. Relevance of AI research – Conference Proceedings

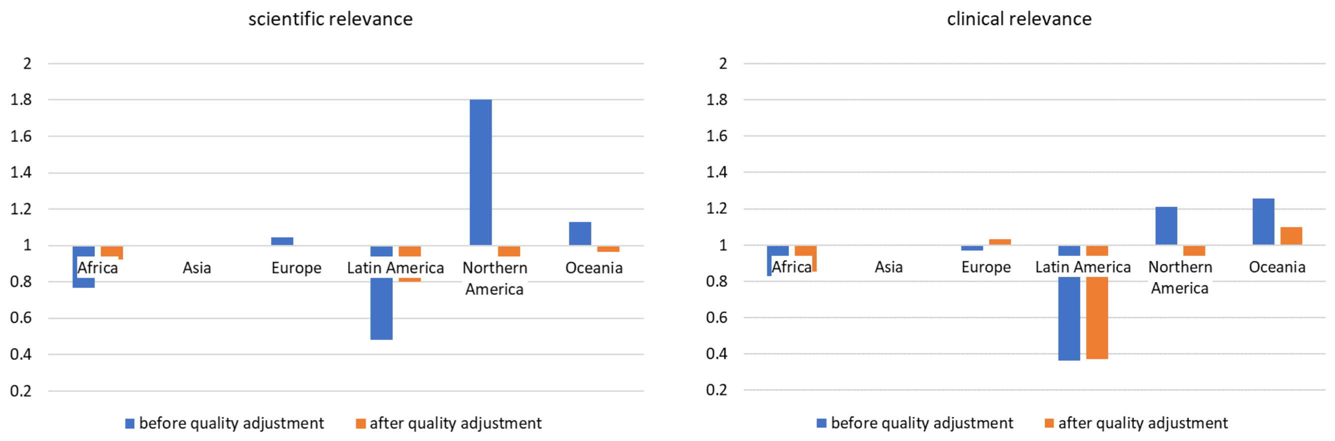

Fig S7.7A | Geography of the relevance of AI life science articles in terms of forward citations in life science research and clinical research. All bars depict incidence rate ratios (IRRs) obtained from negative binomial regressions of citations on dummy variables for the geography of articles published in conference proceedings and recorded in OpenAlex (n = 23,466), with the most productive region, Asia, serving as base category. Blue bars show unadjusted estimates (only accounting for publication year), whereas orange bars also include controls for quality variation across publishing outlets. The left panel considers all citations and the right panel only citations by clinical research.

S7.8A. International collaborations – PubMed

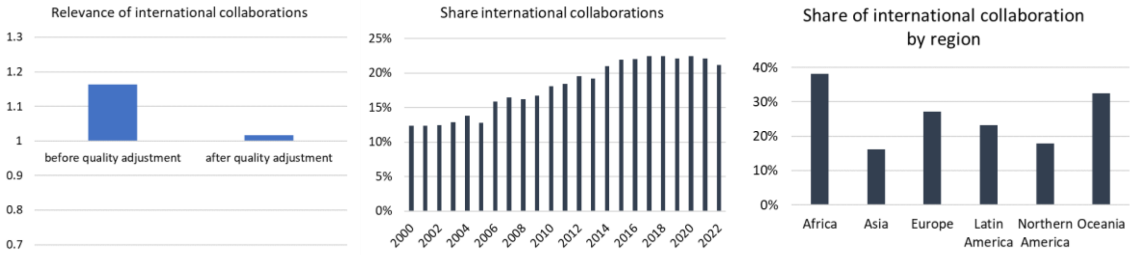

Fig S7.8A. | The effect of international collaboration on scientific and clinical relevance (A); share of international collaborations over time (B); share of international collaborations by region (C). Incidence rate ratios (IRRs) obtained from negative binomial regressions of citations (n= 374,483) and clinical citations (n= 374,440) on a dummy variable for international collaboration on articles recorded in PubMed, accounting for country of lead author, team size, and publication year (A). Percentage share of articles recorded in PubMed with at least two authors affiliated in different countries (n= 374,501) (B). Percentage share of articles recorded in PubMed with at least two authors affiliated in different countries by geographic region (n= 374,501) (C).

S7.8B. International collaborations – Conference Proceedings

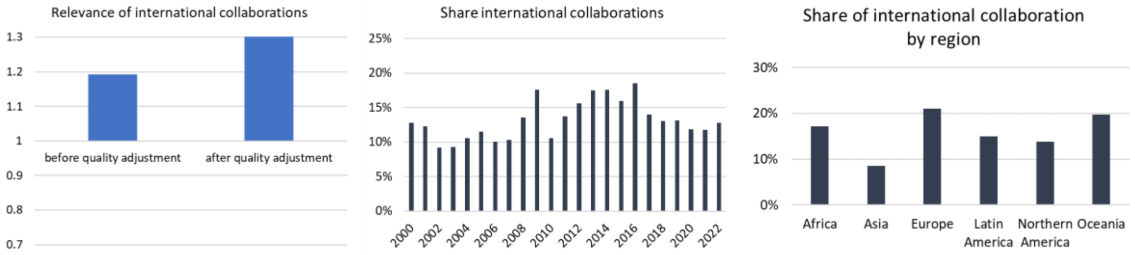

Fig S7.8B. | The effect of international collaboration on scientific and clinical relevance (A); share of international collaborations over time (B); share of international collaborations by region (C). Incidence rate ratios (IRRs) obtained from negative binomial regressions of citations (n= 23,435) and clinical citations (n= 22,132) on a dummy variable for international collaboration on articles published in conference proceedings and recorded in OpenAlex, accounting for country of lead author, team size, and publication year (A). Percentage share of articles published in conference proceedings and recorded in OpenAlex with at least two authors affiliated in different countries (n= 23,466) (B). Percentage share of articles published in conference proceedings and recorded in OpenAlex with at least two authors affiliated in different countries by geographic region (n= 23,466) (C).

S7.9A. Alluvial diagram of international collaborations – PubMed

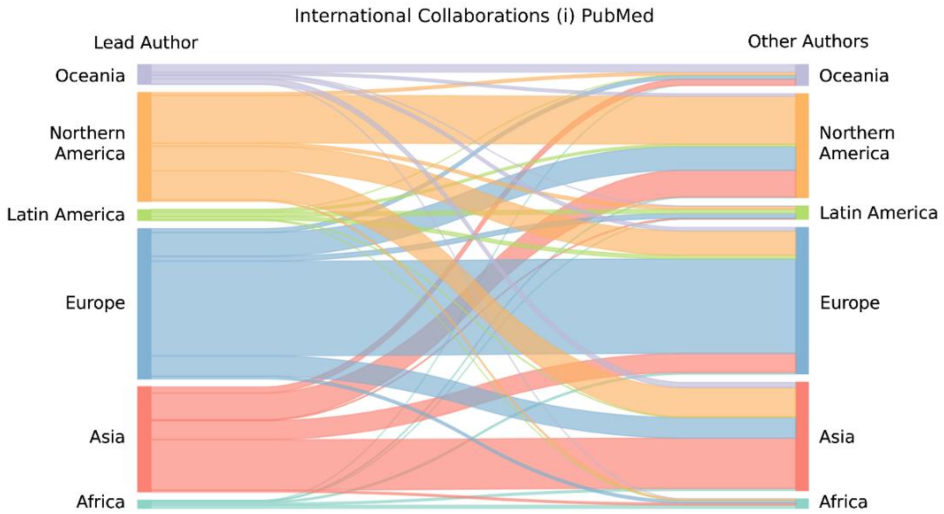

Fig S9.A | Alluvial diagram of international collaborations. Number of dyadic collaborations between authors from different countries, aggregated to the regional level, on articles recorded in PubMed. Dyadic collaborations are counted as co-authorships between a publication's lead author (last author or first author otherwise) and any other author on the author byline that is from a different country. Only international dyads are considered (n = 101,292 dyads).

S7.9B. Alluvial diagram of international collaborations – Conference Proceedings

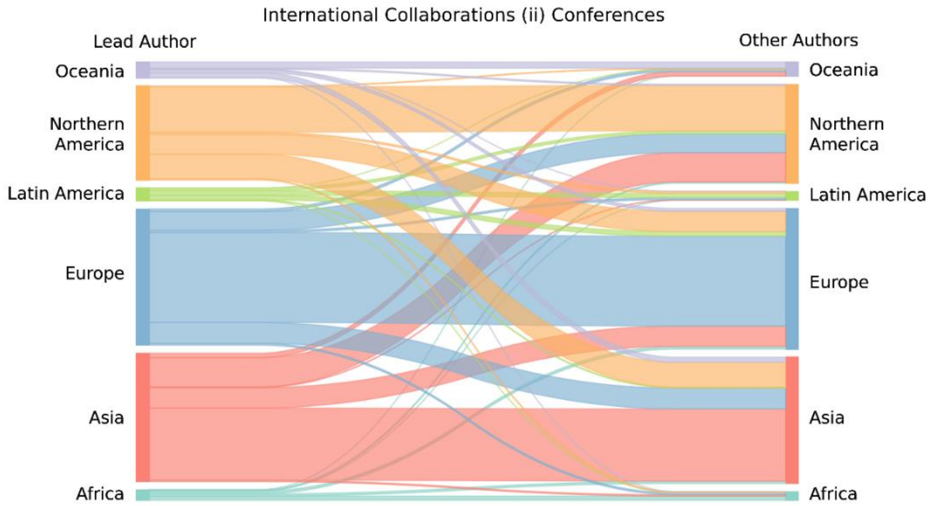

Fig S9.A | Alluvial diagram of international collaborations. Number of dyadic collaborations between authors from different countries, aggregated to the regional level, on articles published in conference proceedings and recorded in OpenAlex. Dyadic collaborations are counted as co-authorships between a publication's lead author (last author or first author otherwise) and any other author on the author byline that is from a different country. Only international dyads are considered (n = 3,966 dyads).

S8 References

1. Wang K, Shen Z, Huang C, Wu C-H, Dong Y, Kanakia A. Microsoft academic graph: When experts are not enough. *Quantitative Science Studies* **1**, 396-413 (2020).

2. OpenAlex. Concpets – OpenAlex Technical Documentation (2024).

3. Baruffaldi S*, et al.* Identifying and measuring developments in artificial intelligence. *OECDiLibrary* (2020).

4. Liu N, Shapira P, Yue X. Tracking developments in artificial intelligence research: constructing and applying a new search strategy. *Scientometrics* **126**, 3153-3192 (2021).
